# Choice and Utility of Pacing Maneuver in Establishing the Mechanism of Supraventricular Tachycardia: A Single Center Experience

**DOI:** 10.4021/cr135w

**Published:** 2012-01-20

**Authors:** Saddam Abisse, Evan Adelstein, Sandeep Jain, Samir Saba

**Affiliations:** a Department of Internal Medicine, University of Pittsburgh Medical Center, USA; b Cardiac Electrophysiology Section, University of Pittsburgh Medical Center, USA

**Keywords:** Supraventricular tachycardia, Mechanism, Pacing maneuvers, Diagnosis

## Abstract

**Background:**

To evaluate the choice and utility of pacing maneuvers in the electrophysiology (EP) laboratory in establishing supraventricular tachycardia (SVT) mechanism.

**Methods:**

We retrospectively examined a cohort of 160 consecutive patients with SVT presenting for invasive EP evaluation to a single center with 8 electrophysiologists. We analyzed the utility of the two most commonly used pacing maneuvers: (1) ventricular entrainment (VE) and (2) His-refractory premature ventricular stimuli (HRPVC) during SVT.

**Results:**

VE was performed in 96 patients: atrial tachycardia (AT) 12, atrioventricular nodal reentrant tachycardia (AVNRT) 66, and orthodromic reciprocating tachycardia (ORT) 18. During VE, AT patients were most likely to have ventriculo-atrial (VA) dissociation (AT 58%, AVNRT 18%, ORT 0%, P < 0.001) and had a tendency towards less SVT termination (AT 0%, AVNRT 9%, ORT 11%, P = 0.19). HRPVCs were delivered in 39 patients: AT 1, AVNRT 24, and ORT 14. Advancement of atrial signal with HRPVC was only observed in ORT (AT 0%, AVNRT 0%, ORT 79%, P < 0.001) and SVT termination was also mostly observed in ORT (AT 0%, AVNRT 4%, ORT 21%, P = 0.33). The overall diagnostic utility of VE was lowest in AT (AT 42%, AVNRT 71%, ORT 83%, P = 0.04), while HRPVC was rarely used in AT. Furthermore, the utilization of maneuvers varied extensively (0% to100%) among the 8 electrophysiologists.

**Conclusion:**

There is great variation in the utilization of pacing maneuvers and their utility in ascertaining the mechanism of SVT. Our results support the fact that discerning AT from AVNRT mechanism remains the most challenging task in SVT diagnosis.

## Introduction

Establishing the mechanism of paroxysmal supraventricular tachycardia (SVT) can be challenging and time consuming, possibly placing patients at increased risk of complications. Several studies have evaluated the diagnostic value of the arrhythmia features and pacing maneuvers [1-4]. It remains unclear, however, which maneuvers have a better diagnostic yield and are most commonly used in a real-life setting. The objective of this study was to evaluate the choice of maneuvers in the electrophysiology (EP) laboratory and estimate their diagnostic yield in determining the mechanism of SVT in a single center with 8 experienced electrophysiologists.

## Methods

### Patient population

Consecutive patient with documented SVT who underwent invasive EP testing and ablation at our institution between January 1, 2008 and December 30, 2009 were included in this analysis. Institutional Review Board approval was obtained prior to any research activities. Patients’ medical records were reviewed and clinical and demographic data were extracted. Baseline EP findings, SVT characteristics, and pacing maneuvers were determined from the patients’ operative notes and EP laboratory tracings.

### Electrophysiology procedure

After obtaining written informed consent, invasive EP testing was performed using standard techniques. Briefly, patients were instructed to discontinue all anti-arrhythmic medications at least 5 half-lives prior to the procedure, which was performed in the fasting state under conscious sedation. Femoral venous access was obtained and quadripolar catheters were positioned in the high right atrium, the region of the His bundle, and at the right ventricular apex. A decapolar catheter was placed in the coronary sinus as needed. Pacing was performed using a digital stimulator. Body surface and intracardiac electrograms were recorded using a commercial recorder (Pruka System, General Electric). SVT was induced using programmed atrial and/or ventricular extrastimuli or burst pacing. Intravenous isoproterenol was used to facilitate induction or maintenance of SVT in 43% of cases. Successful ablation (96%) was defined as the inability to induce, at the end of the procedure, a previously inducible SVT. The mechanism of SVT was determined by the staff electrophysiologist based on data accrued during the EP Study.

### Statistical analysis

Continuous variables are expressed as mean ± SD and were compared using the Kruskal-Wallis test. Mann-Whitney U testing was used to evaluate for statistical significance within groups. Comparisons of nominal variables were performed using Chi-square analysis. All statistical analyses were performed using SPSS (version 11.0, Chicago, IL, USA) statistical software. A p value < 0.05 was considered significant.

## Results

### Patient characteristics

There were 160 consecutive patients (age 50 ± 17 years, mean left ventricular ejection fraction 54 ± 13%, 69 men) with SVT included in the present analysis. Some patients had symptoms associated with the SVT: palpitations (64%), dizziness (14%), or syncope (10%). The patients’ baseline characteristics are shown in Table 1.

**Table 1:** Baseline Patients Characteristic

	Total (n = 160)	AVNRT(n = 84)	AT (n = 29)	ORT (n = 47)	P-value
Age yrs	50.3 ± 17.5	53.0 ± 16.8	59.2 ± 14.4	40.2 ± 15.8	< 0.001*
Female Gender n (%)	91 (57%)	50 (60%)	18 (62%)	23 (50%)	0.41
Race (white) n (%)	143 (89%)	75 (89%)	27 (93%)	41 (87%)	0.34
CAD n (%)	27 (17%)	18 (21%)	7 (24%)	2 (4%)	0.02*
Beta blocker use n (%)	82 (51%)	46 (55%)	17 (59%)	19 (40%)	0.20
Calcium-channel blockers n (%)	28 (18%)	18 (21%)	9 (31%)	1(2.1%)	0.002*
Anti-Arrhythmics n (%)	29 (18%)	18 (21%)	6 (21%)	5 (11%)	0.28
Ejection Fraction (%)	54 ± 13	55 ± 12	47 ± 18	57 ± 8	0.14
Common Symptoms					
Palpitations	94 (64%)	50 (63%)	15 (63%)	29 (66%)	0.09 0.19 0.23
Syncope	15 (10%)	10 (13%)	2 (8%)	3 (7%)	0.09 0.19 0.23
Dizziness	21 (14%)	14 (18%)	0	7 (16%)	0.09 0.19 0.23

Values reported as mean ± SD; * Statistically significant difference between ORT and other two groups.

### Baseline electrophysiology characteristics

The mean baseline cycle length (CL) in sinus rhythm was 846 ± 189 ms and exhibited no significant differences among the 3 arrhythmia groups. Pre-excitation with shorter PR and longer QRS intervals was only evident in the ORT group. The AH interval was significantly longer in the AT group (96 ± 30 ms as compared to the mean for the whole cohort 86 ± 32 ms P = 0.01). The HV interval and atrioventricular (AV) Wenckebach CL were significantly shorter in the ORT group (Table 2).

**Table 2:** Baseline EP Characteristics

	Total	AVNRT	AT	ORT	P-value
CL (ms)	843 ± 189	854 ± 165	779 ± 227	861 ± 202	0.254
PR interval (ms)	151 ± 40	164 ± 35	173 ± 31	116 ± 31	< 0.001^§^
QRS interval (ms)	101 ± 38	88 ± 17	97 ± 24	128 ± 56	< 0.001^§^
QT interval (ms)	398 ± 57	396 ± 41	402 ± 47	400 ± 82	0.105
AH Interval (ms)	86 ± 32	86 ± 29	96 ± 30	81 ± 37	0.01^¥^
HV interval (ms)	42 ± 23	51 ± 18	49 ± 10	21 ± 23	< 0.001^§^
AV Wenckebach (ms)	370 ± 74	375 ± 74	387 ± 76	331 ± 56	0.018^§^
VA wenckebach (ms)	412 ± 114	398 ± 98	478 ± 154	394 ± 114	0.184
Presence of Pre-excitation (%)	20	0	0	79	< 0.001

^§^P-value represents difference between ORT and other two groups, no statistically difference between AT and AVNRT; ^¥^difference between AT and ORT.

### Pacing maneuvers

Among the 160 patients included in this study, 29 had focal atrial tachycardia (AT) from the following locations: high Crista terminalis (n = 10), right atrial free wall (n = 8), septal or posterior right atrium (n = 3), coronary sinus (n = 8), and left atrial source (n = 5); 84 had atrioventricvular nodal reentrant tachycardia (AVNRT): typical AVNRT (n = 77) and atypical AVNRT (n = 7); and 47 had orthodromic reciprocating tachycardia (ORT): right free wall (n = 17), posteroseptal (n = 11), anteroseptal (n = 3), and left lateral (n = 16) accessory pathways. Eleven of these accessory pathways were concealed. Ventricular entrainment (VE) at a cycle length (CL) 10 – 40 ms shorter than SVT was attempted in 96 patients (AT 12, AVNRT 66, and ORT 18). Introduction of His-refractory HRPVCs was performed in 39 patients (AT 1, AVNRT 24, and ORT 14).

### SVT characteristics

The average CL of the SVTs was 351 ± 84 ms for the entire group. The septal ventriculo-atrial (VA) time was significantly shorter in the AVNRT compared to the other groups (252 ± 58 ms, 28 ± 50 ms, and 135 ± 73 ms for the AT, AVNRT, and ORT groups respectively, P < 0.001). AT was most likely to exhibit left bundle aberrancy and most likely to require isoprotenerol for induction and maintenance Eccentric atrial activation during SVT was seen primarily in the ORT and AT groups but not in the AVNRT group (Table 3).

**Table 3:** SVT Characteristics

	Total	AVNRT	AT	ORT	P-value
CL (ms)	351 ± 84	350 ± 70	388 ± 106	325 ± 89	0.10
% Aberrancy					
Left Bundle	29%	25%	67%	20%	0.002
Right Bundle	13%	0	0	60%	
Septal VA interval (ms)	68 ± 86	28 ± 50	252 ± 58	135 ± 73	< 0.001*
AH Interval (ms)	200 ± 113	218 ± 128	146 ± 48	164 ± 47	0.172
HV interval (ms)	64 ± 25	69 ± 27	59 ± 11	48 ± 10	0.470
Isoproterenol required to sustain tachycardia	43%	48%	64%	24%	0.06
Ablation Success (%)	96	99	83	98	0.003
Eccentric atrial activation (%)	20	1	56	65	0.18

CL = cycle length; * Statistically significant difference between all the three groups.

### Sinus rhythm (SR) maneuvers

During SR in the EP lab, electrophysiologists used several maneuvers and characteristics to differentiate the different mechanisms of SVT. The presence of an AH jump’, defined as a 50 ms increase in AH interval for a 10 ms decrease in coupling of an atrial premature stimulation, was most commonly observed during AVNRT. Furthermore, pre-excitation and an extranodal response to para-Hisian pacing during normal sinus rhythm were exclusively seen in ORT cases (Table 4.)

**Table 4:** Pacing Maneuvers During Normal Sinus Rhythm

	% of Total	% Within SVT		
		AVNRT	AT	ORT
Echo beats during premature atrial stimulation	24	85	13	67
AH Jump during premature atrial stimulation	33	87	25	25
Extranodal response to para Hisian pacing	2.2	0	0	100

### Pacing maneuvers during SVT

Ventricular pacing at a CL 10 - 40 ms shorter than the SVT CL was performed in 96 patients. Atrial entrainment was achieved in 68 patients (AVNRT 48, AT 5, and ORT 15). During VE, AT patients were most likely to have VA dissociation and were least likely to have SVT termination. When atrial entrainment was successful, a VAV response was observed in all but one AVNRT patient and in all ORT patients, whereas a VAAV response was seen in all AT patients. The overall success of this maneuver in advancing the diagnosis of SVT mechanism was lowest in AT (Table 5).

**Table 5:** Pacing Maneuvers During SVT and Diagnostic Utility

	Total (n)	AVNRT (n)	AT (n)	ORT (n)
Ventricular pacing at 10 – 40 ms < SVT CL	96	66	12	18
Entrainment	68	48	5	15
• VAAV Pattern		1	5	0
• VAV pattern		47	0	15
Dissociation	19	12	7	0
Termination	8	6	0	2
Diagnostic yield		71%	42%	83%
His Refractory PVC	39	24	1	14
• Entrainment of Atrium		0	0	11
• Termination		1	0	3
Diagnostic Yield		96%	n/a	100%

HRPVCs were delivered in 39 patients (AVNRT 24, AT 1, and ORT 14). Advancing the atrial signal with a HRPVC was only observed in ORT. Termination of SVT was also mostly seen in ORT. The overall utility of this maneuver in advancing the diagnosis of SVT mechanism was high for both ORT and AVNRT (Table 5).

### Physician preference for pacing maneuvers

We also evaluated the use of the different pacing maneuvers based on the treating electrophysiologist, excluding physicians who performed fewer than 5 procedures. Although the distribution of SVT mechanism was similar among all 8 physicians, the choice of pacing maneuver used to establish the SVT mechanism varied significantly ranging at times from 0% to 100% (Table 6).

**Table 6:** Physician Choice of Pacing Maneuver

Physician	AVNRT		AT		ORT	
	V-pacing (%)	PVS (%)	V-pacing (%)	PVS (%)	V-pacing (%)	PVS (%)
1	88.9	33.3	75	25	80	40
2	91.7	45.8	14.3	0	41.7	41.7
3	85.7	0	33.3	0	0	0
4	21.4	0	25	0	0	0
5	87.5	37.5	75	0	42.9	28.8
6	90.9	54.5	0	0	33.3	22.2
7	100	0	0	0	50	50
8	50	50	100	0	25	25
P-value	< 0.001	0.013	0.147	0.543	0.380	0.757

V-pacing: ventricular pacing at 10 - 40 ms < SVT CL; PVS: His-refractory Premature stimulus.

## Discussion

In this study we evaluated the choice and diagnostic utility of pacing maneuvers used to discern the mechanism of SVT in a real-life setting at a medical center with 8 experienced electrophysiologists. Patient characteristics and SVT distribution were consistent with published data [5, 6]. The main findings of this study are as follows: (1) there is great variability, by operator, in the use of different SVT characteristics and pacing maneuvers and in the clinical utility of these maneuvers in establishing the SVT mechanism; and (2) the diagnostic utility of VE and HRPVCs was highest in discerning SVT mechanism in ORT and was least helpful in AT. These data taken together suggest the need for further diagnostic maneuvers to help discriminate between SVT mechanisms, primarily between AT and AVNRT. Standardizing the approach to SVT diagnosis and the introduction of more maneuvers may facilitate reaching a diagnosis more efficiently and may decrease the procedural time.

Currently, the diagnosis of mechanism of SVT is established using several tachycardia features, as well as the response to pacing maneuvers [1-4], however there is a continued interest in incorporating new techniques to help reach an accurate distinction between SVT mechanisms [6, 7]. Sarkosky et al. showed that differential atrial pacing during SVT can be very specific for AT if the arrhythmia persists and the resulting VA intervals after pacing were variable (no VA linking). More recently, our group showed that using simultaneous right atrial and right ventricular pacing helps discriminate AVNRT from AT [8] based on the first return intracardiac electrogram after cessation of pacing. Many other maneuvers have been advanced over the years to help in discerning the SVT mechanism. Enumeration all these maneuvers is beyond the scope of the current manuscript.

Despite new pacing maneuvers being introduced, it appears that in a current real-life practice, most of them are not frequently used. This may reflect a significant difference in the familiarity of physicians with some of these maneuvers or the fact that for most cases, a small subset of more established maneuvers may be sufficient to make the diagnosis. According to our data, the choice of the pacing maneuver was not only dictated by the SVT type but also by the operating physician. We have shown that there were statistically significant differences in the rates of utilization of VE and HRPVCs among 8 electrophysiologists. It is beyond the scope of this manuscript to determine what factors may have influenced the choice of pacing maneuvers. Likely factors may include physicians’ preferences and training but these considerations remain highly speculative.

### Limitations of the study

There are some limitations worth noting in this study. First, it is a retrospective analysis, with inherent limitations and biases. Second, this is a single center experience and its results may not reflect experiences at other institutions, although the current study includes several operators with different training backgrounds. Finally, not all maneuvers were performed in all patients either because of operator preference or because some maneuvers may not be performed in certain SVTs as they are not clinically indicated. This variability in the use of SVT characteristics and diagnostic maneuvers formed the basis of our inter-operator analysis.
